# Administration of Vancomycin at High Doses in Patients with Post Neurosurgical Meningitis: A Comprehensive Comparison between Continuous Infusion and Intermittent Infusion

**Published:** 2018

**Authors:** Morteza Taheri, Simin Dadashzadeh, Shervin Shokouhi, Kaveh Ebrahimzadeh, Masoumeh Sadeghi, Zahra Sahraei

**Affiliations:** a *Department of Clinical Pharmacy, School of Pharmacy, Shahid Beheshti University of Medical Sciences, Tehran, Iran.*; b *Department of Pharmaceutics, School of Pharmacy, Shahid Beheshti Medical University, Tehran, Iran. *; c *Department of Infectious Diseases and Tropical Medicine, Loghman Hakim Hospital, Shahid Beheshti Medical University, Tehran, Iran. *; d *Skull Base Research Center, Loghman Hakim Hospital, Shahid Beheshti University of Medical Sciences, Tehran, Iran.*; e *Department of Epidemiology, School of Public Health, Shahid Beheshti University of Medical Sciences, Tehran, Iran.*

**Keywords:** Vancomycin, Pharmacokinetics, Meningitis, Infusions, Central Nervous System

## Abstract

Poor penetration of vancomycin into Central Nervous System (CNS) can lead to treatment failure. The aim of this study was to evaluate and compare CSF concentration and serum pharmacokinetics of high dose vancomycin by continuous infusion *vs.* intermittent infusion in post neurosurgical meningitis patients. Twenty patients were divided into two groups. Patients in intermittent infusion group received vancomycin as a loading dose of 25 mg/kg over two hours, followed by 25 mg/kg over two hours every 12 h. In the Continuous Infusion group, patients received vancomycin as a loading dose of 25 mg/kg over two hours, followed by 50 mg/kg/day by continuous infusion. In the intermittent infusion group, mean ± SD of serum trough, peak and CSF concentrations were 17.49 ± 2.46 mg/L, 41.33 ± 2.73 mg/L, and 4.83 ± 1.05 mg/L, respectively. Mean of CSF/trough% ratio was 27.39 ± 2.43%. A positive linear correlation was found between the serum trough levels and CSF levels (r = 0.970, *P* < 0.001). In continuous infusion group, mean ± SD of serum and CSF concentrations were 24.76 ± 2.02 mg/L and 6.20 ± 1.31 mg/L respectively. Mean ± SD of CSF/serum% ratio was 24.84% ± 3.54%. The serum and CSF levels revealed positive linear correlation (r = 0.902, *P* < 0.001). The mean of CSF concentration in CI group was 6.20 ± 1.31 mg/L which was significantly higher than II group (4.83 ± 1.05 mg/L, *P* < 0.019). CSF/serum ratio did not show any significant difference between the two groups. Continuous infusion of vancomycin makes it possible to achieve faster and constant target level in serum but did not have any significant effect on the penetration (CSF/Serum ratio) of vancomycin in to the CNS.

## Introduction

Among hospital associated infections, post-neurosurgical meningitis is quite important because of high morbidity and mortality rates. The prevalence rate of post neurosurgical meningitis has been reported from 0.3% to 1.5% in many countries. Hence empiric antimicrobial therapy must be administered promptly (1-3). The pathogenesis of infection has an essential role for selecting the appropriate antimicrobial regimen. Clinicians should consider antimicrobial regimen which consist of vancomycin in combination with cefepime, ceftazidime, or meropenem (4). In addition, corticosteroids, particularly dexamethasone, can reduce cerebral edema and decrease intracranial pressure, decrease risk of hearing loss and other neurologic sequel (5). Although, Vancomycin have bactericidal effect on gram positive pathogens that involved in meningitis but penetration of vancomycin in the CNS is limited by its′ physiochemical properties such as hydrophilicity, relatively large molecular weight and in part because it crosses the blood brain barrier by paracellular pathways (6, 7). Although meningeal inflammation due to infection increase CSF penetration of antibiotics, the administration of dexamethasone decrease meningeal inflammation and intensify the inadequate penetration of vancomycin into CNS (6, 8 and 9). Owing to aforementioned, dosing of vancomycin in meningitis can be challenging for two reasons; first, the concentration of vancomycin in the CSF and second, time to reach target CSF concentration that is critical to decrease neurological sequel and mortality (10). Low CSF concentration of vancomycin and emerge of resistance pathogens can lead to treatment failure (11). Therefore, due to the poor prognosis and low therapeutic response to standard doses of vancomycin in patients with post neurosurgical meningitis, dosage and treatment monitoring have been recently re-evaluated (12-14). To overcome mentioned problem, administration of high dose vancomycin and continuous infusion of vancomycin have been suggested. Continuous infusion of vancomycin is an alternative method that has been preferred due to its′ less variation in serum concentration and achieving a constant bactericidal concentration in serum that may lead to better CSF penetration (12, 15). These studies did not compare intermittent infusion *vs.* continuous infusion method and did not mention exact time of CSF sampling in relation with serum sampling, so judgment on their results is not convincing. There are few studies regarding pharmacodynamics of continuous infusion of vancomycin in human models. Unfortunately, most of these studies have inconclusive findings in determining parameters which predicting patients’ outcomes. The most limitation of these studies is low sample size of patients with a variety of infection types. The present study was accomplished to evaluate and compare CSF concentration and serum pharmacokinetics of high dose vancomycin by continuous infusion *vs.* intermittent infusion in post neurosurgical meningitis patients.

## Experimental

*Patients*

This is a randomized clinical trial that was carried out in intensive care unit (ICU) of Loghman Hakim Hospital, Tehran, Iran between January 2015 and January 2017. The Medical Ethics Committee of Shahid Beheshti University of Medical Sciences (SBMU, Iran) approved the study. The protocol of this clinical trial had been documented in «Iranian Registry of Clinical Trials» (IRCT) with number: IRCT2016071114693N6. Written informed consent form was obtained from responsible caregivers. Twenty patients aged between 18 and 65 years with diagnosis of post-neurosurgical meningitis, were enrolled in the study. These patients were all of eligible cases in this two years period. Also, the sample size of this study was similar to other previous studies (12, 16, 17). Diagnosis were based on the clinical signs and symptoms (*e.g.*, headache, loss of consciousness, fever) plus either positive CSF culture or the presence of at least two criteria of CSF proteins more than 100 mg/dL, glucose concentration less than 50 mg/dL or simultaneously obtained serum glucose value less than 50%, CSF lactate more than 36 mg/dL and elevated White Blood Cells (WBC), usually more than 500 cells/μL (17-19).

**Table 1:** The baseline laboratory data of patients

**Study group**	**Patient**	**Serum Creatinine (mg/dL)**	**Blood Urea Nitrogen (mg/dL)**
II Group	Patient 1	1.1	31
	Patient 2	0.9	35
	Patient 3	1.1	27
	Patient 4	1	59
	Patient 5	1.2	38
	Patient 6	0.9	46
	Patient 7	0.9	73
	Patient 8	1.1	45
	Patient 9	1	28
	Patient 10	1	33
CI Group	Patient 1	1	46
	Patient 2	1.1	19
	Patient 3	1	36
	Patient 4	0.9	33
	Patient 5	1.1	41
	Patient 6	0.9	59
	Patient 7	0.9	35
	Patient 8	0.8	67
	Patient 9	1.1	42
	Patient 10	1	28

II: Intermittent infusion group.

CI: Continuous infusion group.

**Table 2:** Mean level of vancomycin in serum and CSF in the two groups

**Study groups**	**Parameter**	**Sample 1**	**Sample 2**	**Mean ± SD**
II Group	Trough (mg/L)	17.64	17.52	17.49 ± 2.46
	CSF (mg/L)	4.65	5.02	4.83 ± 1.05
	CSF/Trough%	26.41	28.42	27.39 ± 2.43
CI Group	Sample (mg/L)	24.37	25.15	24.76 ± 2.02
	CSF(mg/L)	6.02	6.38	6.20 ± 1.31
	CSF/Sample%	24.48	25.21	24.84 ± 3.54

II: Intermittent infusion group.

CI: Continuous infusion group.

**Table 3:** Pharmacokinetic parameters categorized by the study groups

**Study group**	**Patient**	**Ke (1/h)**	**Half-life (h)**	**Clearance (L/h)**	**AUC (mg × h/L)**
CI Group	Patient 1	0.110	6.300	5.360	559.680
	Patient 2	0.099	7.000	4.716	636.000
	Patient 3	0.090	7.700	4.980	602.400
	Patient 4	0.109	6.357	4.662	643.440
	Patient 5	0.100	6.930	4.284	583.440
	Patient 6	0.092	7.532	3.814	655.440
	Patient 7	0.127	5.456	5.521	543.360
	Patient 8	0.090	7.700	5.359	513.120
	Patient 9	0.100	6.930	4.048	617.520
	Patient 10	0.080	8.662	3.292	683.280
	Mean ± SD	0.099 ± 0.13	7.05 ± 0.89	4.60 ± 0.73	603.76 ± 53.76
II Group	Patient 1	0.085	8.152	4.061	615.550
	Patient 2	0.090	7.700	4.147	602.760
	Patient 3	0.105	6.600	5.677	528.365
	Patient 4	0.110	6.300	4.674	641.760
	Patient 5	0.104	6.663	4.350	689.640
	Patient 6	0.103	6.728	5.590	536.630
	Patient 7	0.100	6.930	6.012	582.095
	Patient 8	0.094	7.372	4.726	634.705
	Patient 9	0.118	5.872	4.962	604.475
	Patient 10	0.091	7.615	4.445	562.320
	Mean ± SD	0.10 ± 0.01	6.99 ± 0.7	4.86 ± 0.68	599.83 ± 49.59

II: Intermittent infusion group.

CI: Continuous infusion group.

**Figure 1:**
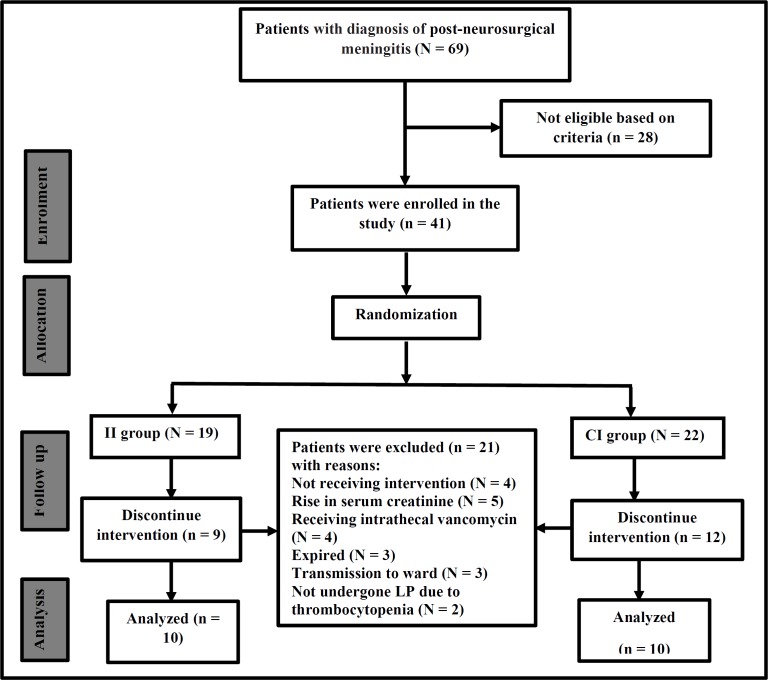
CONSORT flow-chart of the study

**Figure 2:**
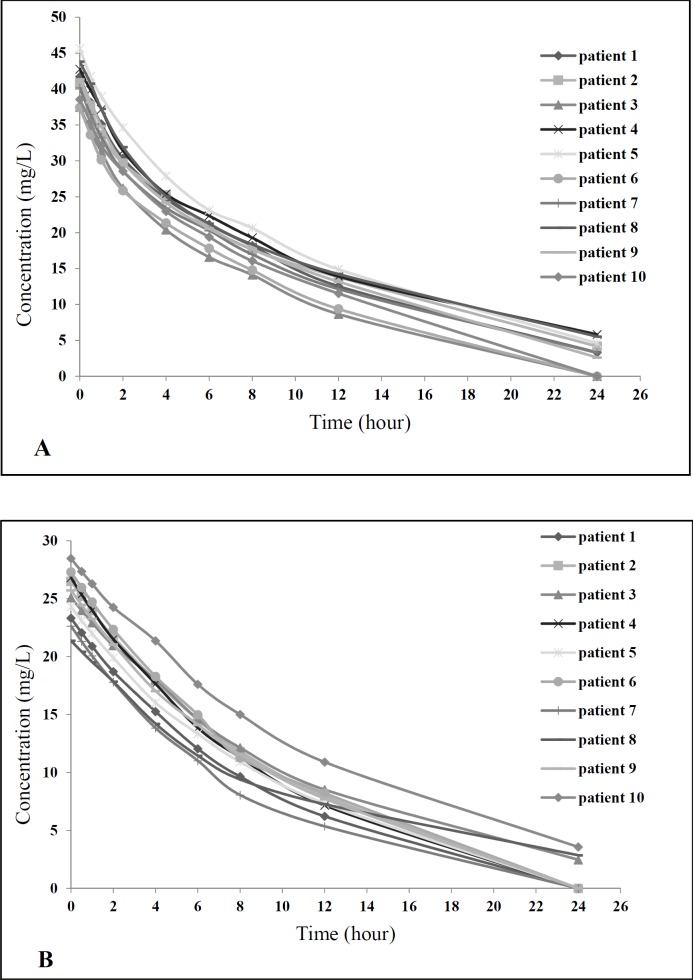
Concentration-time curve in the two study groups (A) Group A and (B) Group B

Exclusion criteria were defined as: pregnancy, chronic kidney disease (creatinine clearance less than 50 mL/min) before treatment, occurring of acute kidney injury (based on AKIN criteria) during treatment (20), vancomycin trough level more than 30 mg/L, receiving intrathecal or intraventricular vancomycin, primary or secondary immune deficiency, patients with solid or hematopoietic transplantation. The patients were assigned into two study groups (Intermittent Infusion (II) group and Continuous Infusion (CI) group) using simple randomization by «RAND» function in excel software. Each study group comprises 10 patients. 

*Dosing Regimen*

All patients had received vancomycin as antibiotic prophylaxis two hours before surgery at a dose of 1 g over an hour. In the II group, patients received vancomycin as a loading dose of 25 mg/kg over two hours, followed by 25 mg/kg over two hours every 12 h by intermittent infusion as maintenance dose and meropenem was administered 2 g over 30 min every 8 h. In the CI group, the patients received vancomycin as a loading dose of 25 mg/kg over two hours, followed by 50 mg/kg/day by continuous infusion as maintenance dose and meropenem was administered as same as II group. Additionally, in both groups, dexamethasone was administered 6 mg every 6 h intravenously for 2 days, with the first dose given just before or concomitant with the first dose of antimicrobial therapy.

*Serum Sampling*

In II group, to determine vancomycin serum concentration, the serum samples were obtained at the baseline before starting vancomycin then to find out trough and peak concentrations of vancomycin, the serum samples were obtained 30 min before and an hour after each maintenance doses, respectively. After achieving steady state concentration, on the days 4 and 8 concomitant with serum trough samples (trough 4 and 8), CSF samples (CSF 1 and 2) were obtained to determine CSF concentration of vancomycin.

Determination and identification of vancomycin serum concentration in CI group was exactly the same as the II group. At first, serum samples were obtained at the baseline before starting vancomycin then in accordance with II group, the serum samples were obtained daily. Like the II group, after achieving steady state concentration, on the days 4 and 8 concomitant with serum samples (serum samples 4 and 8), CSF samples (CSF 1 and 2) were obtained to determine CSF concentration of vancomycin. Additionally, for determining pharmacokinetic parameters of vancomycin, the serial serum samples were obtained at 0, 0.5, 1, 2, 4, 6, 8, 12, and 24 h after starting the last dose of vancomycin in both groups.

*Assessment of Vancomycin Level*

Vancomycin concentration, as a primary outcome, was measured by Fluorescent Polarization Immunoassay (FPIA) method by Cobas Integra apparatus (Roche Instrument Center, Rotkreuz, Switzerland). Prior to the measurement, the method was validated in terms of linearity, precision, and accuracy.

*Pharmacokinetic Analysis*

Pharmacokinetic parameters were calculated by using non-compartmental method. Elimination rate constant (K_e_) was calculated by least squares regression of serum concentration–time data points in the terminal log-linear region of the curve. The elimination half-life (t_1/2_) was calculated as 0.693 divided by K_e_. The area under the serum concentration-versus time curve (AUC) was calculated using the trapezoidal rule with extrapolation to infinity. Clearance (Cl) was calculated by dividing dose over AUC.

*Evaluation of Treatment Efficacy*

To evaluate the efficacy and response to the treatment as secondary outcome, CSF cultures and CSF samples were analyzed and the vital signs and clinical response were monitored. To avoid nephrotoxicity, the serum creatinine was measured daily and Glomerular Filtration Rate (GFR) was calculated then patients with GFR less than 50 mL/min were excluded from the study. CSF concentration and CSF/serum concentration ratio was calculated and compared between two groups to evaluate penetration of vancomycin into the CSF.

*Statistical Analysis*

The descriptive statistics presented and Kolmogorov-Smirnov test used to determination the normality distribution. Consequently, the independed *t*-test was used for parametric variables and mann-whitney U test was used for non-parametric variables. *P*-value less than 0.05 was considered as significance level. The correlation between serum and CSF concentration of vancomycin was examined by Pearson’s correlation coefficient. Data were analyzed using SPSS software version 21 (IBM SPSS Statistics for Windows, Version 21.0. Armonk, NY: IBM Corp).

## Results

During a 2-year period, 69 patients with diagnosis of post-neurosurgical meningitis were screened, 28 patients were excluded according to exclusion criteria and 41 patients were enrolled in the study. During follow of study, 21 patients were excluded as described in Figure 1 and finally 20 patients finished the study. The Consolidated Standards of Reporting Trials (CONSORT) flow-chart of the study are presented in Figure 1.

The mean age ± SD of patients in II group and CI group was 49 ± 7.25 and 48 ± 8.02, respectively. The age was not significantly different between two groups (*P* = 0.773). The sex ratio (Female/Male) also was 1 in II group and 2/3 in CI group. The baseline laboratory data of participants were summarized in Table 1. The reasons of surgery in II group were brain tumor (4 cases), head trauma (2 cases), subarachnoid hemorrhage (2 cases), hydrocephalus (1 case) and drug resistant epilepsy (1 case). Also, in CI group the reasons of surgery were brain tumor (3 cases), drug resistant epilepsy (2 cases), hydrocephalus (2 cases), head trauma (2cases), and subarachnoid hemorrhage (1 case).

In the II group, after 4 to 5 doses, the steady state serum concentration was achieved. At steady state, mean ± standard division (SD) serum trough and peak concentrations were 17.49 ± 2.46 mg/L and 41.33 ± 2.73 mg/L respectively and mean ± SD of CSF concentration was 4.83 ± 1.05 mg/L. A positive linear correlation was found between the serum trough levels and CSF levels (r = 0.970, *P* < 0.001). The mean ± SD value for CSF-to-trough concentration ratio was 27.39% ± 2.43%.

In CI group, after 3 doses, steady state serum concentration was achieved. At steady state, mean ± SD serum concentration of samples 4 and 8 and overall average serum concentration, were 24.37 ± 1.92 mg/L, 25.15 ±2.24 mg/L, and 24.76 ± 2.02 mg/L, respectively. Mean ± SD of CSF concentrations was 6.20 ± 1.31 mg/L. The serum and CSF levels revealed positive linear correlation (r = 0.902, *P* < 0.001). The mean ± SD value for CSF-to-serum concentration ratio was 24.84% ± 3.54%. As shown in Table 2, the mean ± SD of CSF concentration in CI group was 6.20 ± 1.31 mg/L which was significantly higher than the value obtained for the II group (4.83 ± 1.05, *P* = 0.019). However, the CSF/serum ratio did not show any significant difference between the two studied groups. Results of vancomycin concentration in serum and CSF in both groups are summarized in Table 2.

To evaluate the efficacy and response to the treatment, the CSF cultures and CSF samples were analyzed. The most of cultures were negative, in the II group just one patient had positive CSF culture (*Acinetobacter baumannii*) and in the CI group two patients had positive CSF culture (Methicillin Resistant *Staphylococcus Aurous* (MRSA) and *Pseudomonas Aeruginosa*). Biochemical analyses in both groups were associated with trend of treatment. All patients recovered and in spite of high dose of vancomycin in both groups, the therapy was well tolerated. In 5 patients, GFR fell down and were excluded from the study according to exclusion criteria; these patients have received medications (like aminoglycosides) that increased risk of nephrotoxicity.

For calculating vancomycin pharmacokinetic parameters, the serial serum samples were obtained at predetermined times, after the last dose of vancomycin in both groups started. Then pharmacokinetic parameters were calculated as mentioned in the methods. As shown in Table 3, based on the findings related to comparing pharmacokinetic parameters between study groups, there was no statistical significance difference in pharmacokinetic parameters between two groups. Concentration-time curve for all patients in both study groups showed similar pattern (Figure 2).

## Discussion

With increasing the use of vancomycin over last decades and emerging resistance pathogens because of inappropriate dosing especially in CNS infections, controversies regarding the most appropriate way to its administration have been developed (1, 10 and 12). In adults with ventriculitis, CSF penetration ranges from 5% to 10% after intravenous administration, resulting in sub therapeutic levels (21), although some studies suggest that CSF concentrations (up to 22% of serum concentrations) are attained when the meninges are inflamed (22). Due to the poor prognosis and low therapeutic response to standard doses of vancomycin in patients with post neurosurgical meningitis, continuous infusion and high doses of vancomycin have been suggested (22). A few studies have evaluated high dose vancomycin in meningitis, initially studied in children (60 mg/kg/day) and more recently in adults (45 mg/kg/day). Their findings revealed that higher vancomycin doses appear to increase efficacy and penetration into CSF and accelerate response to treatment in acute meningitis (23, 24). Hence, we design the present study to evaluate and compare CSF concentration and serum pharmacokinetics of high dose vancomycin by continuous infusion *vs.* intermittent infusion in post neurosurgical meningitis patients. Our result showed, as it was expected, serum concentration of vancomycin in the CI group was more constant than the II group. Also CSF concentration of vancomycin in the CI group was significantly higher than the II group that may lead to higher bactericidal effect of vancomycin. It is worth mentioning that in spite of administration of equal doses in both groups, a higher CSF concentration was achieved in the CI group. Although CSF concentration of vancomycin in the CI group was significantly higher than the II group, there was no significant differences in CSF/Serum ratios between two groups. As lower fluctuation in serum concentration in CI group, it is reasonable to compare CSF/Serum ratio in CI group vs. CSF/Trough ratio in II group, because trough concentration in II group and mean serum concentration in CI group, both are most stable and lowest concentrations in both groups. In both groups, CSF and serum concentration revealed positive linear correlation, indicating that the higher level of serum concentration leads to the higher CSF levels. It seems that higher CSF concentration in the CI group to be the result of the higher serum concentration. These results showed that continuous infusion did not have any significant effect on the penetration and distribution of vancomycin into the CSF.

The most of studies reported CSF penetration of vancomycin about 30%, but Shokuhi *et al.* in 2014, reported mean CSF/Serum ratio about 80% which is the highest value for the vancomycin CSF/Serum ratio ever reported. They determined vancomycin trough level in serum and CSF of patients with community acquired meningitis. The mean serum trough level and mean corresponding CSF level of vancomycin were 13 mg/L and 11 mg/L, respectively. Although the exact reason for this reported highest ratio is not clear, lack of dexamethasone administration to the patients and low serum vancomycin concentration (13 mg/L) may be more likely reasons, also their measurement method by High Performance Liquid Chromatography (HPLC) was different from the other studies (17). There are few studies that evaluated CSF penetration of vancomycin in continuous infusion method. Albanese *et al.* in 2000, investigated the CSF penetration of vancomycin administered by continuous infusion (50-60 mg/kg/day) in 13 mechanically ventilated patients with different types of infections. CSF/Serum ratio in the meningitis patients was 48% *vs.* 18% in the non-meningitis patients. They concluded that vancomycin penetration in meningitis group is higher than non-meningitis group. Although they used high doses of vancomycin, they didn′t compare their data with intermittent infusion method and number of patients with meningitis were low (7 patients) (12). A clinical trial to show the relations between serum and CSF levels of vancomycin in 14 patients with bacterial meningitis was carried out by Ricard *et al.* in 2007. They showed that adequate levels (mean, 7.9 mg/L) of vancomycin in the CSF was obtained by administration of continuous infusion of high-dose vancomycin. The mean serum concentration of vancomycin was 25.2 mg/L (ranging between 14.2 mg/L and 39.0 mg/L) (16) .Similar to current study results, they found a significant positive correlation between vancomycin concentrations in the serum and CSF (r = 0.68). CSF/Serum ratio in their study was about 30% that is close to our results (24%–27%). They concluded that higher vancomycin doses (60 mg/kg/day) appear to overcome the negative effect of dexamethasone on the vancomycin CSF concentration. Although they achieved higher CSF concentration and CSF/Serum ratio, they used higher doses compared to the present study that may increase the risk of drug toxicity (16). These studies did not compare intermittent infusion versus continuous infusion method in their evaluations and did not determine exact time of CSF sampling in relation with serum sampling. Also these studies have enrolled small populations of patients with a variety of infection types. Hence, judgment on their results is not convincing. Although our results showed faster attainment of target concentrations by continuous infusion of vancomycin, as shown in Table 3, pharmacokinetic parameters in both groups are comparable and very close to each other, indicating that vancomycin pharmacokinetic is not dependent on concentration. Also the method of infusion does not modify the pharmacokinetics of vancomycin. Studies demonstrated that vancomycin activity is dependent on the AUC 24 h/MIC, because of this time-dependent activity, administration of vancomycin in divided doses at shorter intervals increase time of maintaining serum concentration above the MIC (25-27), but our results indicate that administration of vancomycin by continuous infusion may be a better way to maximize this time. Although it would be better to evaluate AUC and pharmacokinetic parameters of vancomycin in CSF, the calculation of this parameter in CSF needs multiple times of LP and CSF sampling that is impossible unless the patients have CSF shunt, hence we suggest to design a study to evaluate AUC of vancomycin in CSF in patients with CSF shunting. In spite of a high dose of vancomycin in both groups, the therapy was well tolerated and no adverse effect was observed, this maybe because of slow infusion rate, appropriate concentration of vancomycin solution, and sufficient hydration of patient. Other studies that used high doses of vancomycin didn′t observe drug toxicity (13, 28) .A meta-analysis suggested that continuous infusion is associated with a significantly lower risk of nephrotoxicity when compared with intermittent infusion of the drug (29). Clinical successes with administering vancomycin via continuous infusion have been reported for different infections, for example: catheter infection, pneumonia, osteomyelitis, and septic arthritis and continuous infusion reduced the period of bacteremia in relation to infection (12). Continuous infusion of vancomycin is cheaper and logistically more convenient, that achieves target vancomycin concentrations faster, results in less variability in serum vancomycin concentrations, and requires less therapeutic drug monitoring (30, 31). But a review by Dimondi *et al*. stated that the pharmacodynamics profiles between continuous infusion and intermittent infusion of vancomycin were comparable and continuous infusion therapy did not significantly improve the efficacy of vancomycin in the treatment of invasive MRSA infections (27). In the current study in both groups biochemical parameters were associated with the trend of treatment and there was no difference in clinical impact between two groups. A reason for this conclusion may be administering equal high doses of vancomycin in both groups. Also, our study was limited by its small sample size due to conducting in a single center and low prevalence of disease.

## Conclusion

Continuous infusion of vancomycin makes it possible to achieve faster and constant target level in serum but did not have any significant effect on the penetration (CSF/Serum ratio) of vancomycin, although lead to higher CSF concentration of vancomycin. It seems that higher CSF concentration by continuous infusion of vancomycin is the result of constant and higher serum concentration. High-dose, continuous infusion of vancomycin may be considered in patients not responding to standard dosing methods and impairment of vancomycin penetration into CSF could be overcome by increasing the dosage of vancomycin.
